# Exploring the association between community-level factors and health literacy using multilevel analysis

**DOI:** 10.1186/s12889-025-25724-3

**Published:** 2025-11-29

**Authors:** Inhyung Cho, Sung-il Cho

**Affiliations:** Department of Public Health, Graduate School of Public Health, Seoul National University, 1 Gwanak-ro, Gwanak-gu, Seoul, 08826 Republic of Korea

**Keywords:** Health literacy, Community-level factors, Social capital, Health resources, Multilevel analysis

## Abstract

**Background:**

This study aimed to examine how community-level factors—particularly health resources and social capital—affect health literacy (HL) among residents in South Korea.

**Methods:**

We used data from the 2021 Community Health Survey and the Korean Community Health Status Indicators, incorporating both individual- and community-level variables. Key predictors of HL were first identified using elastic net regression to address multicollinearity. Subsequently, we applied Multilevel (or Hierarchical) logistic regression models using SAS version 9.4, and accounted for clustering at the community level, to assess the combined influence of individual characteristics and community-level conditions.

**Results:**

Multilevel analysis showed that individual characteristics including age, gender, income, occupation, and education were significantly associated with HL. Among the health resource variables, greater availability of healthcare professionals per 100,000 people (OR = 0.95, 95% CI = 0.94–0.99) and sports facilities (OR = 0.96, 95% CI = 0.94–0.98) were associated with higher HL. On the other hand, higher rates of unmet medical needs predicted lower HL (OR = 1.02, 95% CI = 1.01–1.04). Social capital indicators, such as mutual aid, religious participation, and social activities were also positively, yet marginally, linked to HL.

**Conclusions:**

HL is shaped by both personal and community-level factors. Strategies to improve HL should simultaneously combine efforts to expand health infrastructure with measures to reduce social inequalities. Policymakers should prioritize vulnerable populations and strengthen community-based support systems that enhance access to and engagement with health information.

**Supplementary Information:**

The online version contains supplementary material available at 10.1186/s12889-025-25724-3.

## Introduction

Health literacy (HL) refers to an individual’s ability to “access, understand, and use information in ways that promote and maintain health” for themselves, their families, and their communities [1]. High HL is essential for improving public health, achieving better individual health outcomes, and reducing health disparities [2, 3]. Limited HL is consistently associated with negative health outcomes, including poorer doctor–patient communication, inappropriate healthcare utilization, and delayed diagnoses [4–6]. Individuals with low HL often lack health knowledge and struggle with chronic disease management [7], especially older adults [8]. In addition, HL directly and indirectly associated with self-rated health (SRH), a key indicator of both perceived and actual health status [9].

The role of individual-level determinants of HL—such as age, education and income—is well established [10–12]. However, the primary relationship of the broader community-level context with HL and related health outcomes is not yet fully recognized [10–12].

HL scholars have emphasized that HL should be considered not only an individual attribute but also a societal and community-level issue [13, 14]. However, existing research has largely focused on individual predictors of HL [10–12], leaving a gap in understanding how local health resources and social capital relate to the acquisition and application of HL.

In the literature on regional health determinants, community-level characteristics are often conceptualized across multiple domains, such as clinical care, social and economic factors, the physical environment, and health behaviors [15]. Among these, health resources refer to the availability and accessibility of healthcare and related facilities, whereas social capital encompasses the networks, trust, and engagement that facilitate collective action for health. Although physical activity infrastructure (e.g., sports centers) is sometimes classified under the physical environment, in this study, it is included in the health resources domain to reflect its functional role in promoting health, as spaces that encourage physical activity are related to healthier lifestyles and improved mental well-being [16].

Community-level socioeconomic factors also are associated with health outcomes [17–19]. Health-related resources and infrastructure can are related to better population health beyond the association with individual behaviors [20–22]. Social capital, including strong social ties and support systems, also is related to improved health outcomes by buffering stress and promoting health-seeking behaviors [23–25]. Many studies address how community-level or social factors are related to health outcomes such as disease incidence, mortality, and self-rated health [22, 25–27], and access to health information [28–30]. However, relatively few studies have directly examined the association of community-level characteristics with HL itself.

This study aims to address this gap by examining how community-level factors—particularly health resources and social capital—are associated with HL. We hypothesize that these contextual characteristics have an independent association with HL beyond those of individual-level variables. Identifying the pathways through which community-level conditions are related to HL can offer new insights for public health policy and intervention design. Effective public health promotion requires not only targeting individual behaviors but also considering the community-level environments in which people live [26].

## Methods

### Participants

**Fig. 1:**
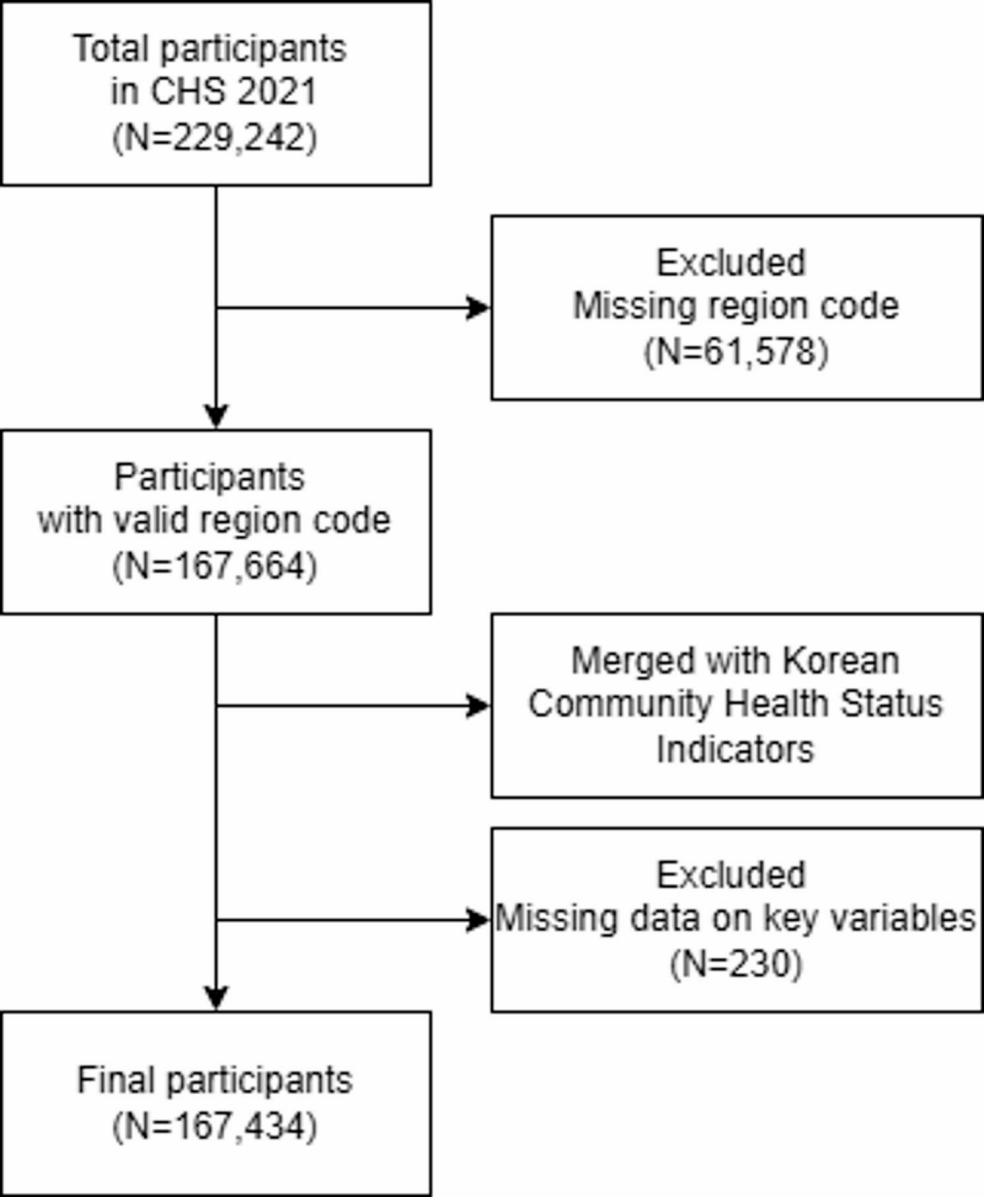
Process of participants selection

This study relies on data from the 2021 Community Health Survey (CHS), an annual nationwide survey conducted by the Korea Disease Control and Prevention Agency (KDCA) since 2008, and from the Korean Community Health Status Indicators, which provide regional-level data for 278 administrative areas in Korea. Several data-cleaning procedures were performed. First, participants with missing regional codes were first excluded (*n* = 61,578) because these codes were required to link community-level indicators. After merging the two datasets, the merged dataset (*n* = 167,664) was screened for missing values in key study variables, leading to the exclusion of 230 participants with incomplete data. The final analytical sample consisted of 167,434 individuals across 256 districts and cities. A flow diagram summarizing the sample selection and data-cleaning process is presented in Fig. 1.

### Variables

The dependent variable, HL, was defined based on two questions from the 2021 CHS regarding participants’ understanding of oral and written health information. Respondents who answered “somewhat difficult” or “very difficult” to both questions (“How difficult is it for you to understand information that doctors, nurses, and other health professionals tell you?” and “In general, how difficult is it for you to understand written health information?”) were categorized as having low HL and coded as 1 (Low HL), while others were coded as 0 (High HL).

Individual-level covariates included age, sex, monthly household income, occupation, education level and marital status. Occupational classifications were coded by interviewers according to the Korean Standard Classification of Occupations, and were regrouped into four categories: “White-collar” (managers, professionals and related workers, and clerical workers), “Service/Sales“(service workers and salespeople), “blue collar” (workers in agriculture, forestry, and fisheries, technicians and related workers; equipment and machinery operators; and manual laborers), and “Others” (military personnel and the unemployed).

The chronic condition variable was derived from responses to the questions, ‘Have you ever been diagnosed with the following medical conditions by a doctor?’ regarding hypertension and diabetes. Accordingly, participants were categorized into three groups: no condition, one condition, and two conditions. Community-level variables were derived from the Korean Community Health Status Indicators, which contains data on 278 Korean regions. We selected variables from 4 major categories: health outcomes, the physical environment, health care systems, and demographic characteristics. Conceptually, these were regrouped into two broader categories: health resources (HR) and social capital (SC).

HR variables included the number of doctors, pharmacists, dentists, oriental medicine doctors, nurses, clinics, and social workers; the availability of sports facilities (e.g., gyms, stadiums, swimming pools); and contextual indicators such as the number of fast-food restaurants, bars, and tobacco retailers; the healthcare facility utilization rate; and the unmet medical needs rate. The variable ‘healthcare professionals’ was calculated by summing the number of doctors, pharmacists, dentists, oriental doctors, and nurses, and then standardizing the sum per 100,000 people.

The SC variables reflected neighborhood-level engagement and trust, including mutual trust, mutual aid, and monthly participation in religious, social, leisure, and charity activities. These variables indicate the extent to which residents trust each other, offer assistance in events such as weddings or funerals, and regularly participate in community life.

For regions that did not match during the merging of the CHS and DB datasets, subregion information was combined to reprocess the data (e.g., Cheonan in Chungcheongnam Province, Jeonju in Jeollabuk Province, Masan and Changwon in Gyeongsangnam Province).

### Statistical analysis

Descriptive analyses were performed to examine differences in HL between groups and chi-square tests were conducted to compare the proportions of low HL and high HL groups.

Due to the large number of predictors and potential multicollinearity, we applied elastic net regression for variable selection. Elastic net is a regularization technique that combines the penalties of both LASSO (L1) and ridge (L2) regression, enabling it to handle correlated predictors more effectively while also performing variable selection [31]. This method was particularly suitable for identifying the most relevant predictors of HL from a broad range of variables, including those representing community-level HR and SC.

Following variable selection, we conducted a multilevel analysis to further explore the association of community-level factors with HL while controlling for individual-level covariates. This hierarchical modeling approach accounts for the nested data structure (individuals within regions), enabling the estimation of both within-region (individual-level) and between-region (community-level) associations. This approach was essential for assessing the extent to which the variance in HL could be attributed to community-level contexts beyond individual characteristics. Results are expressed as odds ratios (ORs) with 95% confidence intervals (CIs).

All tests were two-tailed, and no adjustments for multiple comparisons were made, as the analyses were exploratory. Adjusted odds ratios (aOR) from multilevel models are reported, controlling for individual- and applicable community-level covariates. A *p*-value of < 0.05 was considered statistically significant. All analyses were conducted using SAS version 9.4.

### Ethical approval

This study utilized publicly available, de-identified secondary data and was granted an exemption from ethical review by the Institutional Review Board of Seoul National University (IRB No. E2403/004–007). All procedures were performed in accordance with the Declaration of Helsinki and relevant guidelines and regulations.

### Data availability

The datasets analyzed in this study are publicly available from the Community Health Survey conducted by the Korea Disease Control and Prevention Agency and can be accessed at https://chs.kdca.go.kr.

## Results

Table 1 shows that the average age of the participants was 52.8 years. Participants in their 60 s constitute the largest group (35,306, 21.09%), whereas those in their 20 s constitute the smallest group (18,905, 11.29%). Slightly more than half of the women (89,157, 53.20%). The most common monthly household income group was 2–4 million Korean won (29.89%). The military personnel and unemployed individuals constituted the largest occupation group (35.30%), followed by the blue collar group (27.67%). The percentage of students with a bachelor’s degree or higher was 37.85%, and the percentage of high school graduates was 36.06%. More than two-thirds of the respondents were married (68.64%).

**Table 1:** Sociodemographic characteristics of the study population

		Health Literacy (*N* = 167,434)			
		High HL (%)	Low HL (%)	Total	*p*-value
Age group	20 s	15,359(81.24)	3,546(18.76)	18,905	< 0.0001
	30 s	16,334(78.37)	4,507(21.63)	20,841	
	40 s	23,004(79.08)	6,086(20.92)	29,090	
	50 s	26,313(76.53)	8,068(23.47)	34,381	
	60 s	24,411(69.14)	10,895(30.86)	35,306	
	70 s	14,597(50.49)	14,314(49.51)	28,911	
Gender	Male	57,983(74.07)	20,294(25.93)	78,277	< 0.0001
	Female	62,035(69.58)	27,122(30.42)	89,157	
Monthly household income	< 2 million KRW	25,574(58.35)	18,255(41.65)	43,829	< 0.0001
	2–4 million KRW	36,454(72.84)	13,592(27.16)	50,046	
	4–6 million KRW	31,346(77.55)	9,073(22.45)	40,419	
	≥ 6 million KRW	26,644(80.4)	6,496(19.60)	33,140	
Occupation	White collar	32,403(82.7)	6,777(17.30)	39,180	< 0.0001
	Service/Sales	17,350(76.00)	5,478(24.00)	22,828	
	Blue collar	30,654(66.17)	15,673(33.83)	46,327	
	Others*	39,611(67.02)	19,488(32.98)	59,099	
Education level	< High school	21,751(49.81)	21,920(50.19)	43,671	< 0.0001
	High school	45,550(75.43)	14,834(24.57)	60,384	
	≥ Bachelor’s degree	52,717(83.18)	10,662(16.82)	63,379	
Marital status	Married	83,451(72.62)	31,470(27.38)	114,921	< 0.0001
	Not married	36,567(69.63)	15,946(30.37)	52,513	

*Others: military personnel and unemployed (include students and homemakers)

The proportion of low HL was lower than that of high HLs across all age groups. Households with a monthly income less than 2 million Korean Won has the highest proportion of low HL (41.65%), and the white-collar group has the lowest low HL (17.30%). Similarly, individuals with less than a high school education had the highest proportion of low HL(50.19%).

The elastic net analysis revealed that, among the major categories of community health-related factors (health outcomes, the physical environment, the healthcare system, and sociodemographic characteristics), five HR variables and five SC variables were correlated with HL.

Table 2 summarizes all the variables selected. The HR variables included the density of health professionals (per 100,000 population), the density of social workers (per 100,000 population), the density of sports facilities (per 100,000 population), the annual healthcare facility utilization rate, and the annual unmet medical needs rate. The SC variables included mutual trust, mutual aid, religious activities, social activities, and charity activities.

**Table 2:** Variable selection outcomes from elastic net regression

Health Resource		Social Capital	
Selected variable	Coefficient Estimates	Selected variable	Coefficient Estimates
Density of health professionals	−0.0202	Mutual trust	−0.0045
Density of social workers	0.0148	Mutual aid	0.007
Density of sports facilities	−0.0167	Religious activities	−0.0121
Annual healthcare facility utilization rate	0.0123	Social activities	−0.007
Annual unmet medical needs rate	0.0245	Charity activities	0.0119

Table 3 shows the results of our multilevel analyses. Model 1 is a null model that does not include independent variables. Model 2, which includes only individual factors, suggests that the likelihood of low HL tends to decrease with age. Compared with individuals in their 20 s, the likelihood of low HL was 1.06 times lower for those in their 60 s (95% CI = 1.00–1.12, *p* = 0.037) and 1.8 times lower for those in their 70 s (95% CI = 1.70–1.90, *p* < 0.001). Females were 1.13 times more likely to have low HLs than males (95% CI = 1.11–1.16, *p* < 0.001). Additionally, higher income levels were associated with a lower likelihood of low HL. For example, individuals in the highest household income group were less likely to have low HL than those in the lowest income group (OR = 1.22, 95% CI = 1.17–1.27, *p* < 0.001). The likelihoods were similar for those earning 2–4 million Korean won (OR = 1.08, 95% CI = 1.04–1.12, *p* < 0.001) and those earning 4–6 million Korean won (OR = 1.05, 95% CI = 1.01–1.09, *p* = 0.008). Compared with white-collar workers, blue-collar workers were 1.26 times more likely to have low HL (95% CI = 1.21–1.31, *p* < 0.001), whereas service/sales workers were 1.06 times more likely to have low HL (95% CI = 1.01–1.10, *p* = 0.014). Educational status also played a significant role: individuals with the highest level of education were less likely to have low HL than those with a high school diploma (OR = 3.94, 95% CI = 3.79–4.11, *p* < 0.001) and those with less than a high school education (OR = 1.55, 95% CI = 1.50–1.60, *p* < 0.001).

**Table 3:** Multilevel logistic regression results for the factors associated with health literacy

	Low Health Literacy(OR, 95% CI)					
	Model 1^a^	Model 2	Model 3	Model 4	Model 5	Model 6
Age group (Ref: 20 s)						
30s		1.48(1.4–1.56)***			1.5(1.42–1.59)***	1.49(1.41–1.57)***
40 s		1.32(1.25–1.39)***			1.33(1.26–1.4)***	1.31(1.25–1.38)***
50 s		1.17(1.11–1.23)***			1.18(1.12–1.25)***	1.17(1.11–1.23)***
60 s		1.06(1–1.12.12)*			1.09(1.03–1.15)**	1.06(1.01–1.12)*
70 s		1.8(1.7–1.9)***			1.85(1.74–1.96)***	1.81(1.7–1.91)***
Gender (Ref: Male)						
Female		1.13(1.11–1.16)***			1.14(1.11–1.17)***	1.14(1.11–1.16)***
Monthly household income (Ref: ≥6 million)						
< 2 million KRW		1.22(1.17–1.27)***			1.16(1.12–1.21)***	1.16(1.12–1.21)***
2–4 million KRW		1.08(1.04–1.12)***			1.02(0.99–1.06)	1.03(1–1.07.07)
4–6 million KRW		1.05(1.01–1.09)**			0.94(0.91–0.98)**	0.95(0.92–0.99)**
Occupation (Ref: White collar)						
Service/Sales		1.06(1.01–1.1)*			1.05(1.01–1.1)*	1.06(1.01–1.11)*
Blue collar		1.26(1.21–1.31)***			1.25(1.2–1.3)***	1.26(1.21–1.31)***
Others		1.15(1.11–1.2)***			1.15(1.1–1.19)***	1.15(1.11–1.19)***
Education level (Ref: ≥ Bachelor’s degree)						
< High School		3.94(3.79–4.11)***			3.96(3.79–4.13)***	3.96(3.8–4.13)***
High School		1.55(1.5–1.6)***			1.55(1.5–1.6)***	1.55(1.5–1.6)***
Marital status (Ref: Married)						
Not married		1.16(1.13–1.19)***			1.17(1.13–1.2)***	1.16(1.13–1.19)***
Chronic disease indicators (Ref: No)						
Has 1		1.09(1.06–1.12)***			1.09(1.06–1.12)***	1.09(1.06–1.12)***
Has 2		1.14(1.09–1.2)***			1.14(1.09–1.2)***	1.14(1.09–1.2)***
Health Resource						
Density of health professionals			0.98(0.96–1.96)*		0.95(0.94–0.99)*	
Density of social workers			1.02(1.01–1.03)*		1.01(0.99–1.02)	
Density of sports facilities			0.98(0.93–1.04)		0.96(0.94–0.98)*	
Healthcare facility utilization rate			1.01(1.01–1.01)***		0.95(0.92–1.01)*	
Unmet medical needs rate			1.03(1.01–1.04)***		1.02(1.01–1.04)***	
Social capital						
Mutual trust				1(0.99–1.99)		1(1–1.01.01)
Mutual aid				1.01(1–1.01.01)***		1(0.99–1.99)*
Religious activities				0.99(0.98–1.98)**		0.99(0.98–1.98)*
Social activities				0.99(0.99–1.99)**		0.99(0.98–0.99)***
Charity activities				1.01(1–1.03.03)		1.02(1–1.04.04)*
Region-level random variance	0.125	0.130	0.097	0.099	0.119	0.112
*p*-value^b^	< 0.0001	< 0.0001	< 0.0001	< 0.0001	< 0.0001	< 0.0001
ICC (%)	3.66	3.81	2.85	2.91	3.5	3.3
AIC	196423.4	180,753	185294.5	194488.3	170161.7	178896.9

*OR* Odds Ratio, *CI* Confidence Interval, *ICC* Intraclass Correlation Coefficient, *AIC* Akaike Information Criterion

a Model 1 is the null (empty) model including only a random intercept for region

b *p*-values correspond to the significance of the region-level random variance

* *p* < 0.05; ** *p* < 0.01; *** *p* < 0.001

Model 3, which analyzes only the community-level HR variables, indicates that individuals living in areas with a higher density of healthcare professionals have a significantly lower probability of low HL (OR = 0.98, 95% CI = 0.96–1.96, *p* = 0.040). Individuals living in areas with a higher density of social workers tend to have low HL (OR = 1.02, 95% CI = 1.01–1.03, *p* = 0.047). The probability of low HL increases significantly with higher annual health institution utilization rates (OR = 1.01, 95% CI = 1.01–1.01, *p* < 0.001) and higher unmet medical need rates (OR = 1.03, 95% CI = 1.01–1.04, *p <* 0.001). Conversely, the density of sports facilities does not appear to be significant.

Model 4, which focuses on community-level SC variables, shows that HL was significantly lower in areas with high rates of religious (OR = 0.99, 95% CI = 0.98–1.00, *p* = 0.001) and social activities (OR = 0.99, 95% CI = 0.99–1.00, *p* = 0.005). The probability of low HL in areas with higher rates of mutual aid, which refers to collective support during times of bereavement or need, was 1.01 (95% CI = 1.00–1.01, *p* < 0.001).

The results of Model 5, which includes both individual- and community-level HR factors, exhibit patterns similar to those of Model 2. Notably, individuals in the highest household income group are more likely to have low HL than those with a monthly household income of 4–6 million Korean won (OR = 0.94, 95% CI = 0.91–0.98, *p* = 0.003). Individuals living in areas with a higher density of health professionals had a slightly lower probability of having low HL (OR = 0.95, 95% CI = 0.94–0.99, *p* = 0.048). The density of sports facilities showed a similar result (OR = 0.96, 95% CI = 0.94–0.98, *p* = 0.043). As expected, a higher unmet medical need rate significantly increases the probability of low HL (OR = 1.02, 95% CI = 1.01–1.04, *p* < 0.001), as expected.

Model 6, which includes both individual- and community-level SC factors, shows that the probability of low HL is significantly lower for individuals residing in areas with higher rates of religious (OR = 0.99, 95% CI = 0.98–1.00, *p* = 0.020) and social activities (OR = 0.99, 95% CI = 0.98–0.99, *p* < 0.001).

In the null model, the intraclass correlation coefficient (ICC) is very low (0.0366), indicating relatively small community-level variation in HL. While there are some differences in HL across regions, individual-level variables are more influential in explaining HL outcomes.

## Discussion

This study aimed to identify community-level factors associated with HL by integrating large-scale data from the 2021 Community Health Survey and the Korean Community Health Status Indicators. While previous studies have focused primarily on individual-level sociodemographic factors (e.g., age, education, income) [32, 33], our study offers novel insights by emphasizing the significant role of community-level HR and SC in shaping HL.

Notably, the number of healthcare professionals and the unmet medical need rates were identified as key predictors of HL. These findings indicate that, beyond individual characteristics, community-level structural resources are related to residents’ ability to access, understand, and utilize health information. The observation that a greater number of medical professionals positively affects HL aligns with previous studies [34–36]^,^ demonstrating that the availability of medical professionals is crucial for improving population HL. Additionally, higher rates of unmet medical needs were correlated with lower HL—supporting and expanding upon earlier findings [37, 38] concerning the relationship between healthcare accessibility and health outcomes.

A novel and somewhat unexpected finding was the observation that, when only community-level factors were examined, a greater number of social workers was associated with lower HL. Although social workers are often assumed to support HLs through their roles in social welfare, this result may reflect their concentration in socioeconomically disadvantaged areas where HL tend to be lower. As this study used a cross-sectional design, causal interpretations are limited. However, this finding raises important questions about the role and focus of social workers, who may prioritize welfare support over direct health education. It also highlights the need for stronger integration of health communication and education strategies into social welfare services.

This study confirmed the positive association between sports facilities and promoting HL, particularly when both individual- and community-level factors were considered. Although the number of sports facilities alone was not significant in the community-level-only model, it became significant in the multilevel model, indicating that interactive environments such as gyms or group exercise classes may facilitate the exchange of health-related knowledge. Additionally, economic affluence, which often correlates with the density of sports facilities [11, 39], may indirectly contribute to higher HL.

With respect to social capital, participation in religious and social activities was positively associated with HL. These findings are consistent with those of previous studies [40] and suggest that communal spaces not only offer social support but also serve as informal venues for health information exchange. Such interactions can strengthen individuals’ ability to verify and share health information, which are both essential components of HL.

To our knowledge, this is one of the few studies that simultaneously examine both HR and SC variables at the community level via a multilevel modeling framework. The key contributions of this study are as follows: First, it empirically demonstrates that the community-level context matters in shaping HL, even after controlling for individual factors. Second, it identifies specific HR and SC indicators (e.g., number of medical professionals, number of sports facilities, unmet medical needs rate, mutual aid, and religious/social participation) that are related to HL. Third, it uncovers new patterns, such as the counterintuitive link between social worker density and lower HL, which merits further investigation.

Our findings highlight that community-level resources, such as healthcare accessibility and opportunities for social participation, play a significant role in shaping HL. This aligns with previous research, which has demonstrated that HL is influenced not only by individual capacities but also by broader social and contextual conditions. For example, Sentell et al. [41] highlighted the influence of community-level contexts on HL, underscoring the importance of structural and environmental conditions in HL research. Similarly, Sørensen et al. [42], in their systematic review and integrated conceptual model, argued that HL should be understood as both an individual capability and a contextual outcome shaped by societal resources, health systems, and civic engagement. Together, these insights support the interpretation that our findings reflect a broader, internationally recognized understanding of HL as embedded in both personal and community environments.

Nevertheless, this study has several limitations. The use of self-reported HL measures may not accurately capture individuals’ actual competencies. The cross-sectional design also limits conclusions about causality. Despite these limitations, the study’s strengths lie in its large, nationally representative sample and the application of multilevel modeling to assess both individual- and community-level associations. This dual-level approach provides a more nuanced understanding of how local environments relate to HL and highlights the importance of investing in both health infrastructure and social networks at the community level. Variables related to various chronic diseases are known to be associated with HL; however, due to the limited scope of the survey, only two conditions, namely hypertension and diabetes, were included in the analysis.

Nevertheless, this study is subject to several limitations. The use of self-reported HL measures may not accurately capture individuals’ actual abilities. The cross-sectional design also limits conclusions about causality. Furthermore, variables related to various chronic diseases are known to be associated with HL; however, due to the limited scope of the survey, only two conditions(namely hypertension and diabetes) were included in the analysis. Despite these limitations, the study’s strengths lie in its large, nationally representative sample and the application of multilevel modeling to assess both individual- and community-level associations. This dual-level approach provides a more nuanced understanding of how local environments relate to HL and highlights the importance of investing in both health infrastructure and social networks at the community level.

Taken together, our findings support the hypothesis that both individual characteristics and contextual factors, including health resources and social capital, play a significant role in understanding patterns of community HL. The results highlight that improving HL cannot be accomplished solely by enhancing health infrastructure; rather, it requires multifaceted strategies that also address socioeconomic disparities and strengthen community engagement. Effective interventions should actively target vulnerable populations through customized education and communication approaches. By recognizing HL as a product of both personal and structural conditions, this study calls for policies that integrate healthcare, social capital, and equity-focused planning to foster resilient and health-literate communities. Future research should further explore the mechanisms through which community-level environments influence people’s ability to access, understand, and apply health information in their daily lives.

From a public health perspective, our findings highlight the importance of investing not only in individual education programs but also in community infrastructure that fosters HL. Strengthening healthcare access, supporting local organizations, and enhancing opportunities for civic participation can serve as cost-effective strategies to reduce health disparities. Additionally, strengthening social capital through initiatives that build trust and encourage mutual aid within neighborhoods further contributes to sustainable health improvements. Policies should recognize HL as both a health outcome and a prerequisite for equitable healthcare utilization, positioning it as a central priority in health promotion and health equity agendas.

## Conclusion

This study demonstrated that individual- and community-level factors, particularly healthcare resources and social capital, are important correlates of HL in Korea. By applying multilevel modeling to nationally representative data, we showed that HL gaps are embedded not only in individual characteristics but also in the broader community context. These findings highlight the need for policies that go beyond individual education programs to strengthen community resources, particularly healthcare resources, and expand opportunities for social participation. Future research should examine specific associative mechanisms and conduct cross-national comparisons to deepen our understanding of how the community context relates to HL across diverse settings.

## Supplementary Information

Supplementary Material 1.

## Data Availability

The datasets analyzed in this study are publicly available from the Community Health Survey conducted by the Korea Disease Control and Prevention Agency and can be accessed at https://chs.kdca.go.kr.
