# Supplementary material for: Exploring the association between community-level factors and health literacy using multilevel analysis
Source: BMC Public Health. 2025 Nov 29;26:51. doi: 10.1186/s12889-025-25724-3 (PMC12772004; doi:10.1186/s12889-025-25724-3)
Supplement: Supplementary file 1 — Supplementary Material 1. [file 12889_2025_25724_MOESM1_ESM.docx]

**Supplement Table**

TABLE A. Distribution of occupations by original CHS occupational categories.

|  | **N** | **%** |
| --- | --- | --- |
| Managers | 3543 | 2.12 |
| Professionals and related workers | 18220 | 10.88 |
| Clerical workers | 17417 | 10.4 |
| Service workers | 13176 | 7.87 |
| Sales workers | 9652 | 5.76 |
| Agriculture, forestry, and fisheries | 14177 | 8.47 |
| Technicians and related workers | 7948 | 4.75 |
| Operators, machine operators, and assemblers | 8015 | 4.79 |
| Workers engaged in manual labor | 16187 | 9.67 |
| Military personnel | 565 | 0.34 |
| Unemployed and economically inactive | 58534 | 34.96 |
